# Supplementary material for: Adgrg6/Gpr126 is required for compact wall integrity and establishing trabecular identity during cardiac trabeculation
Source: Nat Commun. 2026 Feb 7;17:1484. doi: 10.1038/s41467-026-69292-5 (PMC12886893; doi:10.1038/s41467-026-69292-5)
Supplement: Supplementary file 1 — Supplementary Information [file 41467_2026_69292_MOESM1_ESM.pdf]

Supplementary data

Adgrg6/Gpr126 is required for compact wall integrity and establishing trabecular identity during cardiac trabeculation

Swati Srivastava[1,8], Felix Gunawan[2,9,10], Silvia Vergarajauregui[1,3], Alessandra Gentile[2,11,12], Miriam Angeloni[4], Sarah C. Petersen[5,13], Stefan Günther[6], Fulvia Ferrazzi[4,7], Didier Y.R. Stainier[2], Felix B. Engel[1,3,*]

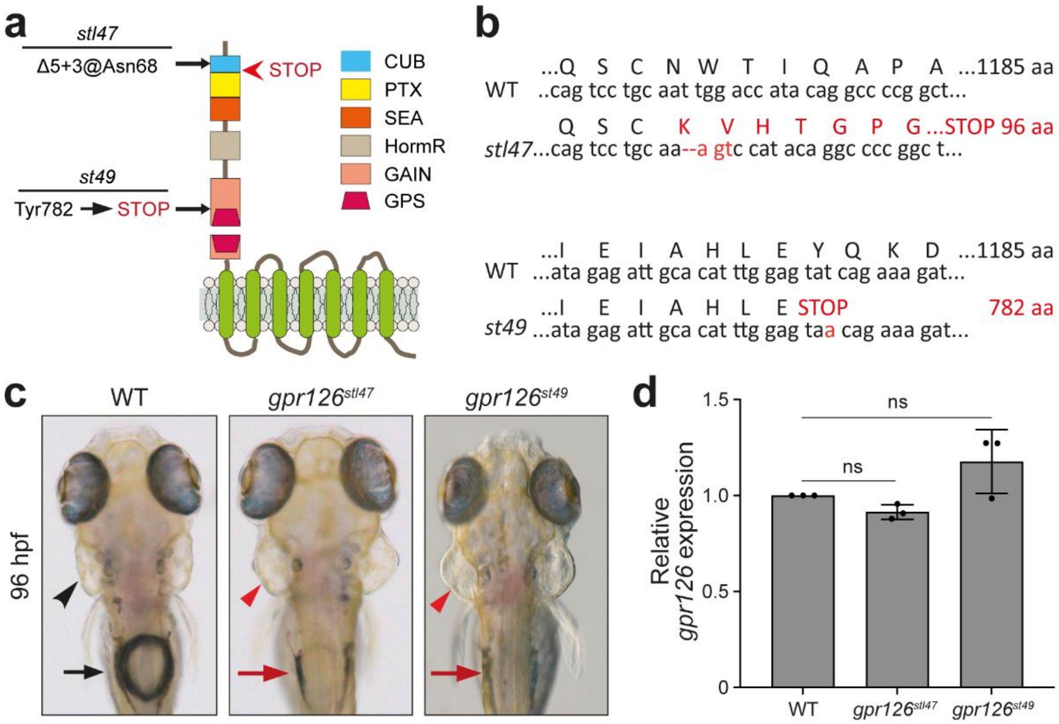

Figure S1. ***gpr126***^{stl47} **and** ***gpr126***^{st49} **mutants exhibit no nonsense-mediated RNA decay**
(a) Schematic representation of zebrafish Gpr126 indicating changes encoded by *stl47* and *st49* alleles. The red arrowhead indicates the STOP codon encoded by *stl47* allele.
(b) Sequence comparison of WT vs. *stl47* allele and WT vs. *st49* allele. WT gpr126 encodes for an 1185 amino acid (aa) protein. The *stl47* allele carries a Δ5+3 indel, predicted to encode for a 96 aa truncated protein. The *st49* allele carries a point mutation that converts an asparagine-encoding codon into a stop codon resulting in a truncation at aa 782.
(c) Dorsal views of WT, *gpr126*^{stl47}, and *gpr126*^{st49} larvae at 96 hours post fertilization (hpf). Mutants exhibit puffy ear and swim bladder inflation defects. n = 5 independent experiments. Black arrowhead: normal ear; black arrow: inflated swim bladder; red arrowhead: puffy ear; red arrow: failure of swim bladder to inflate.
(d) qPCR expression data of *gpr126* in 96 hpf hearts from WT, *gpr126*^{stl47}, and *gpr126*^{st49} larvae. WT vs. *gpr126*^{stl47}: p = 0.503. WT vs *gpr126*^{st49}: p = 0.1186 (one way ANOVA with Dunnett's multiple comparisons test). Data are mean ± SD; n = 3; ns: not statistically significant.

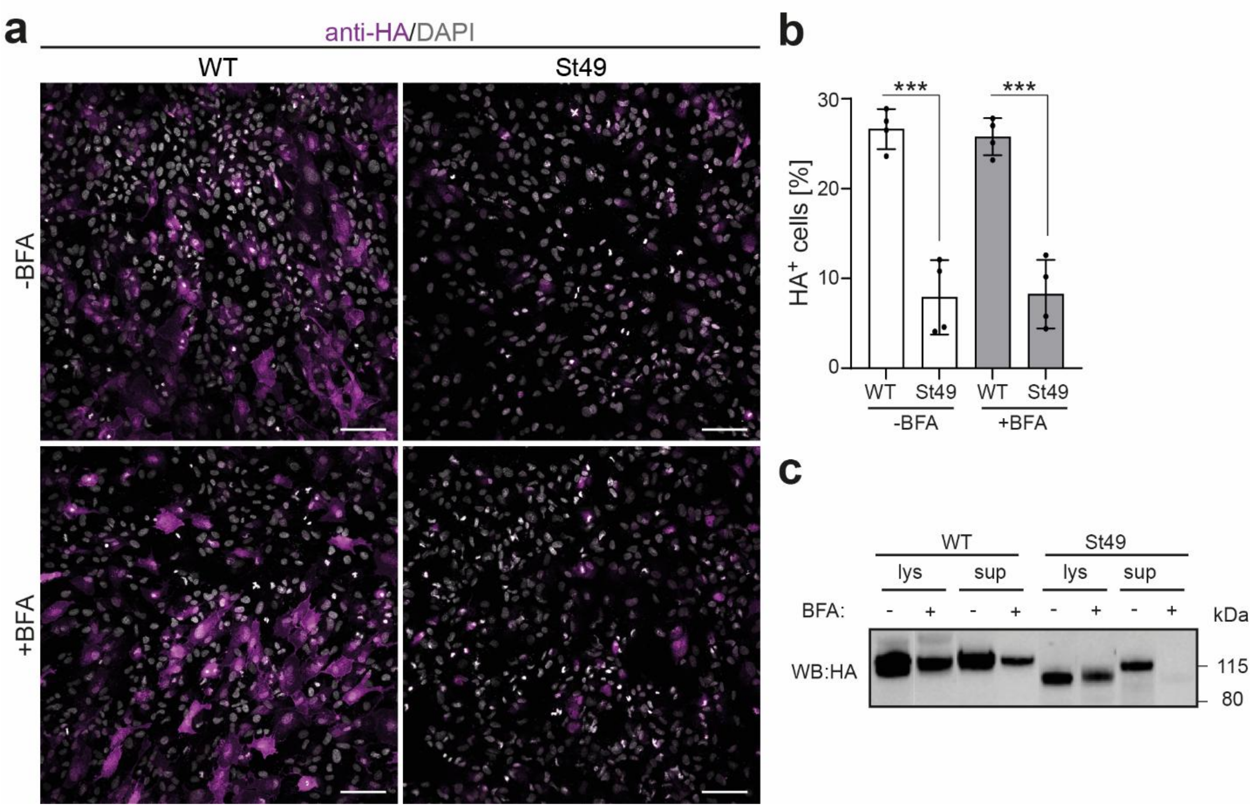

Figure S2. NTF$^{\Delta GPS}$ is secreted in cells transfected with St49 construct.
(a) Representative images of ARPE-19 cells transfected with HA-gpr126-GFP (WT) and HA-gpr126^{st49}-GFP (St49) stained for HA (purple). Nuclei were visualized with DAPI (grey).
Scale bars: 100 µm.
(b) Quantification of percentage of HA-positive cells. n = 4 fields. WT vs. St49, with BFA: p = 0.0002. WT vs. St49, without BFA: p = 0.0002 (two-tailed unpaired student's t-test). Data are mean ± S.D. ***: $p < 0.001$.
(c) Representative western blot (WB) analysis of lysates (lys) and supernatant (sup) from control and brefeldin-A (BFA)-treated ARPE-19 cells transfected with HA-gpr126-GFP (WT) and HA-gpr126^{st49}-GFP (St49) using antibodies against HA; as indicated. n = 3 independent experiments. Note, the order of the WB lanes has been altered to fit the order in Fig. 1f (see source data for the original blot).

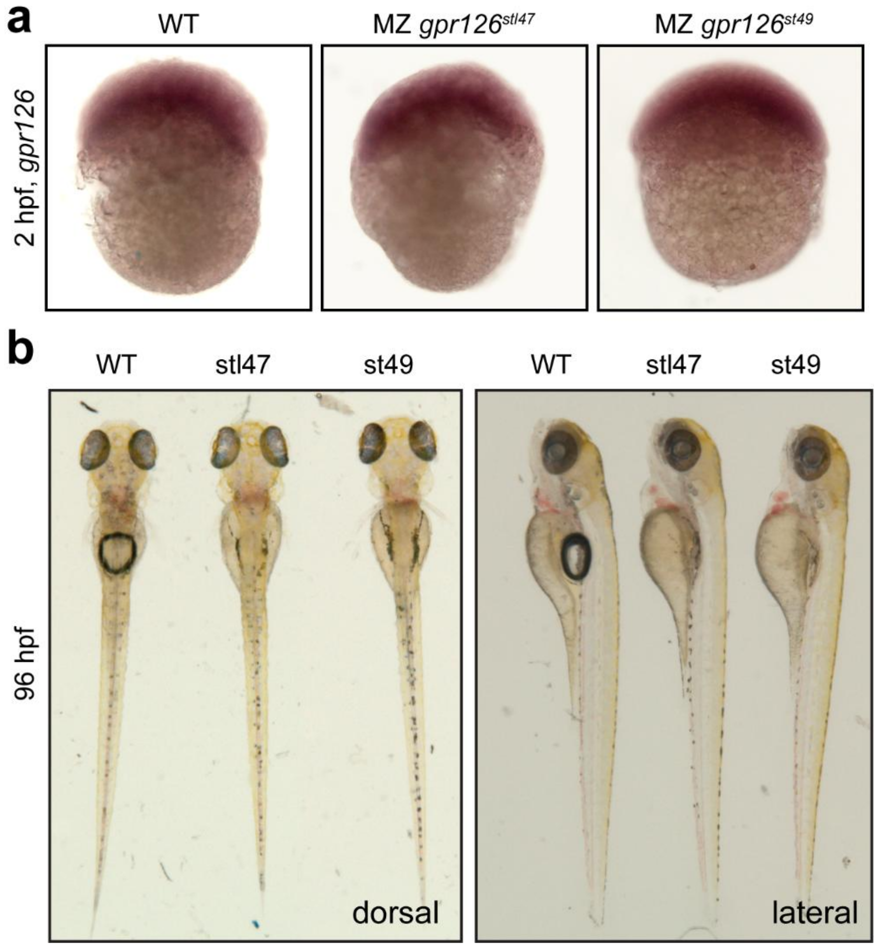

Figure S3. MZ gpr126 mutants exhibit normal gross morphology and have detectable levels of maternal *gpr126* mRNA.

(a) Whole mount in situ hybridization analysis of *gpr126* in WT, maternal zygotic (MZ) $gpr126^{stl47}$, and MZ $gpr126^{st49}$ larvae at 2 hours post fertilization (hpf). *gpr126* show the same level of expression in the WT as well as mutant larvae. *n* = 5 biological replicates.

(b) Dorsal and lateral views of WT, $gpr126^{stl47}$, and $gpr126^{st49}$ larvae at 96 hpf exhibiting similar gross morphology. *n* = 3 independent experiments.

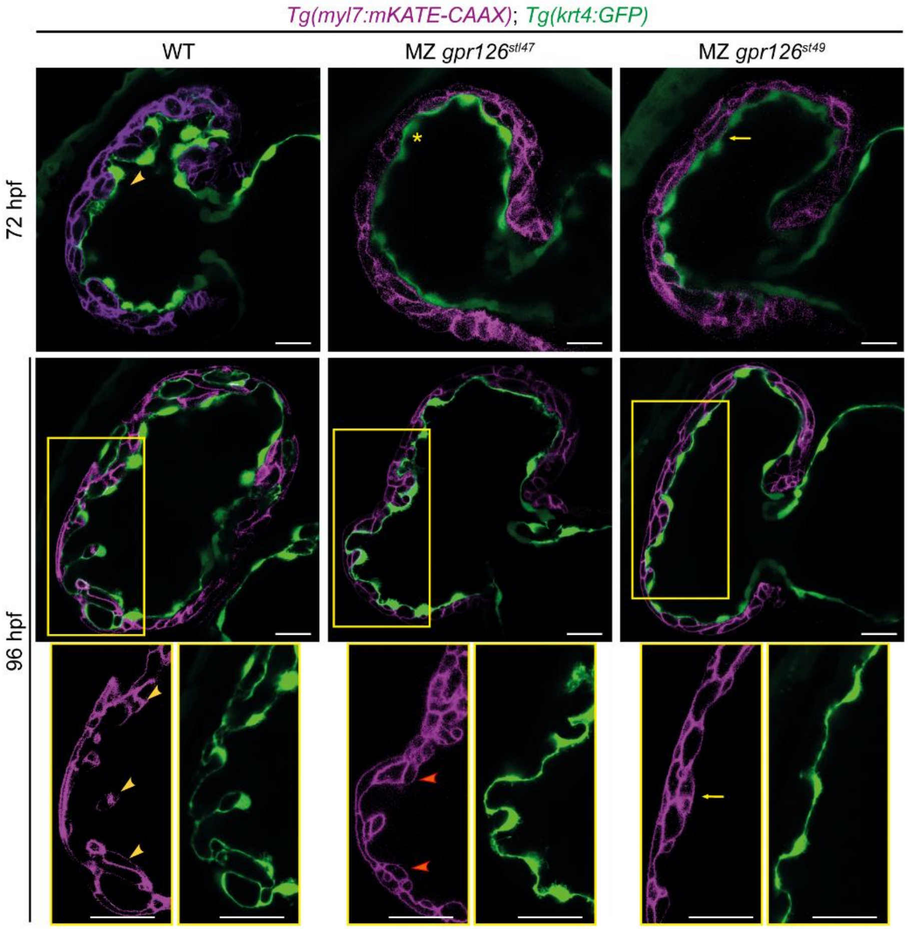

Figure S4. Gpr126 mutants exhibit no obvious endocardial defects.
Confocal images (mid-sagittal sections) of 72 and 96 hours post fertilization (hpf) hearts of WT, maternal zygotic (MZ) *gpr126*^{stl47}, and MZ *gpr126*^{st49} larvae crossed into *Tg(myl7:mKATE-CAAX)*; *Tg(krt4:GFP)*^{sqet33} background. *n* = 8/genotype. Yellow arrowheads: trabeculae. Orange arrowheads: shorter trabeculae. Asterisks: absence of trabeculae. Arrows: multilayered ventricular wall.
Scale bars: 20 µm.

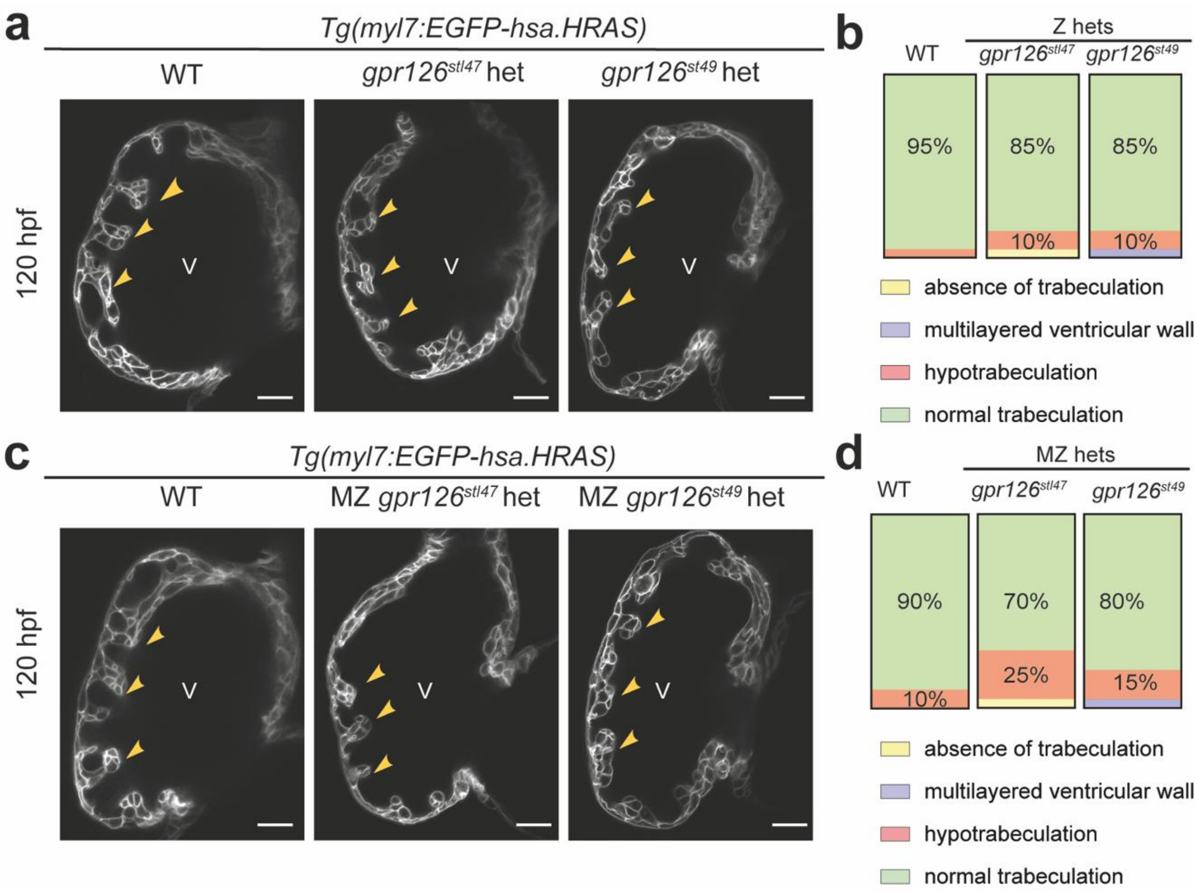

Figure S5. Heterozygous Z and MZ *gpr126^{stl47}* and *gpr126^{st49}* larvae do not exhibit trabeculation defects.

(a) Representative confocal images (mid-sagittal sections) of hearts of WT, heterozygous zygotic (Z het) *gpr126^{stl47}*, and Z het *gpr126^{st49}* larvae crossed into *Tg(myl7:EGFP-hsa.HRAS)* background at 120 hours post fertilization (hpf). Arrowheads: trabeculae, V: ventricle. Scale bars: 20 µm.

(b) Quantification of trabeculation phenotypes in 120 hpf WT, Z hets *gpr126^{stl47}* and Z hets *gpr126^{st49}* larvae. *n* = 20.

(c) Representative confocal images (mid-sagittal sections) of hearts of WT, maternal zygotic (MZ) het *gpr126^{stl47}*, and MZ het *gpr126^{st49}* larvae crossed into *Tg(myl7:EGFP-hsa.HRAS)* background at 120 hpf. Yellow arrowheads: trabeculae, V: ventricle. At: atrium. Scale bars: 20 µm.

(d) Quantification of trabeculation phenotypes in 120 hpf WT, MZ hets *gpr126^{stl47}*, and MZ hets *gpr126^{st49}* larvae. *n* = 20.

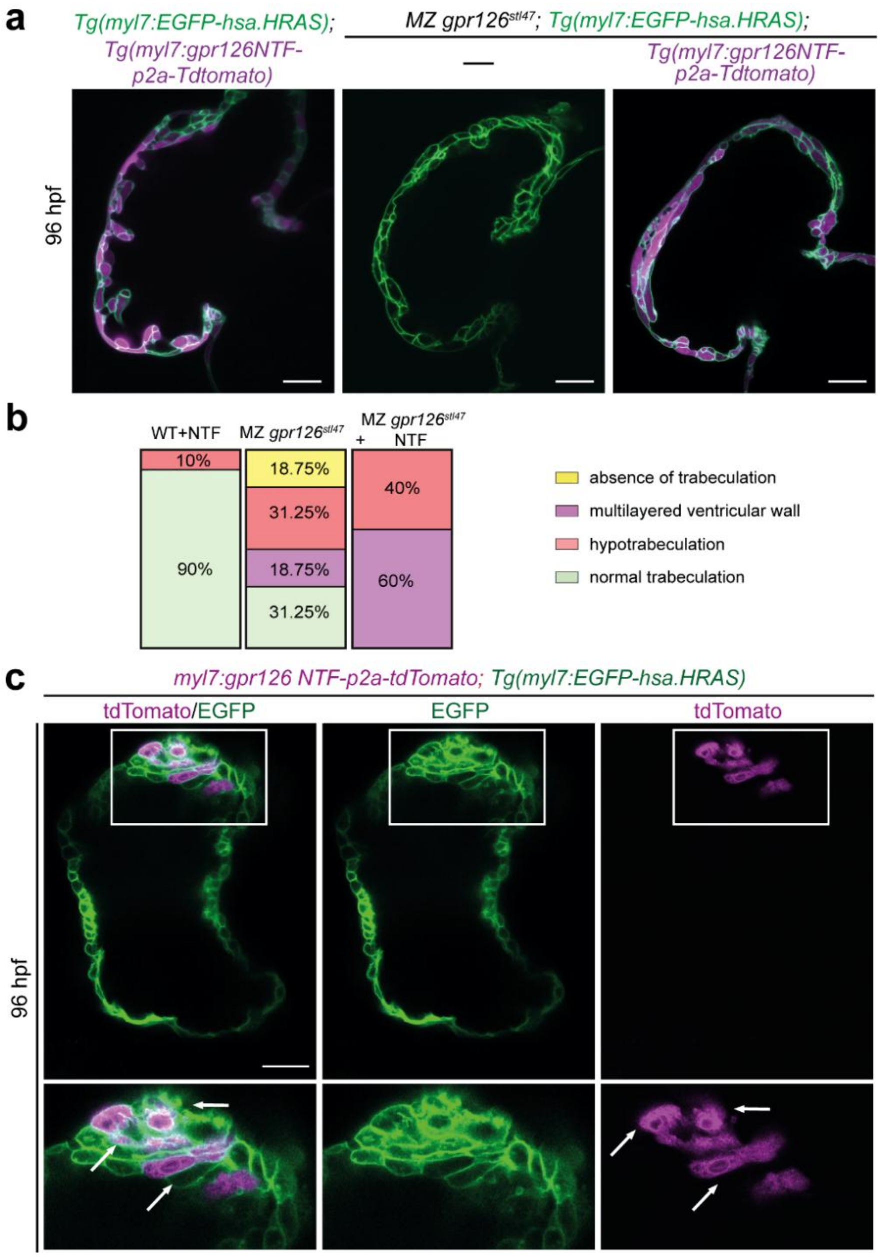

Figure S6. Myocardial-specific NTF overexpression induces cardiomyocyte multilayering in MZ *gpr126^{stl47}* mutants.

(a) Confocal images (mid-sagittal sections) of 96 hours post fertilization (hpf) *Tg(myl7:EGFP-hsa.HRAS),* maternal zygotic (MZ) *gpr126^{stl47}; Tg(myl7:EGFP-hsa.HRAS)* and MZ *gpr126^{stl47}; Tg(myl7:EGFP-hsa.HRAS)*; *Tg(myl7:gpr126NTF-p2a-tdTomato)* hearts (*n* = 10, *n* = 16 and *n* =10, respectively). Scale bars: 20 µm.

(b). Quantification of trabeculation phenotypes in 96 hpf *Tg(myl7:EGFP-hsa.HRAS),* MZ *gpr126^{stl47}; Tg(myl7:EGFP-hsa.HRAS)* and MZ *gpr126^{stl47}; Tg(myl7:EGFP-hsa.HRAS)*; *Tg(myl7:gpr126NTF-p2a-tdTomato)* hearts.

(c) Confocal images (mid-sagittal sections) of 96 hpf *Tg(myl7:EGFP-hsa.HRAS*) hearts injected with a myocardial-specific NTF overexpression construct *(myl7:gpr126 NTF-p2a-tdTomato)* at the single cell-stage. Mosaic overexpression of NTF in WT cardiomyocytes leads to local multilayering (5/8) which is outlined by a white box and magnified. Arrows: NTF overexpressing cardiomyocytes. Scale bars: 20 µm

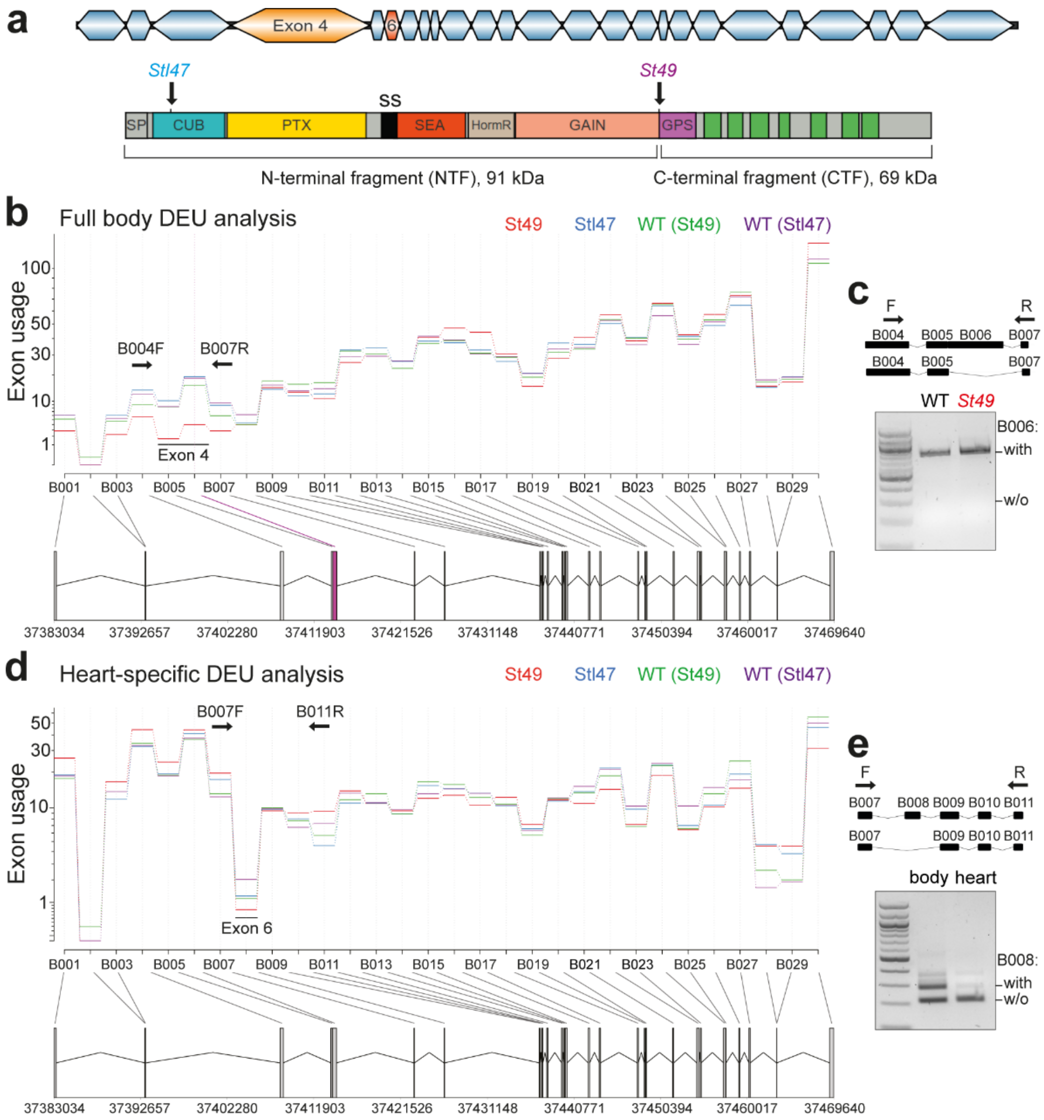

Figure S7. Alternatively spliced gpr126 isoforms do not compensate for non-functional transcripts in the heart.

(a) Schematic representation of Gpr126 protein indicating exon position of the canonical transcript isoform adgrg6-204 and the known splice sites (S). Schematics were generated with Illustrator for Biological Sequences 2.0.

(b) Differential exon usage (DEU) analysis in 72 hours post fertilization (hpf) WT, maternal zygotic (MZ) *gpr126^{stl47}*, and MZ *gpr126^{st49}* larvae. Top panel: exon usage coefficients of each exon counting bin (B) in each sample. Bottom panel: flattened gene model. In purple: B with significant differential exon usage. F: forward primer. R: reverse primer.

(c) RT-PCR analysis of full body WT and MZ *gpr126^{st49}* RNA using primers spanning exon counting bin (B) 004 to B007 indicating no differentially usage of exon 4.

(d) Differential exon usage analysis in 96 hpf WT, MZ *gpr126^{stl47}*, and MZ *gpr126^{st49}* larval hearts. Top panel: exon usage coefficients of each exon counting bin (B) in each sample. Bottom panel: flattened gene model.

(e) RT-PCR analysis of full body and heart-specific RNA of WT larvae using primers spanning B007 to B011 indicating differentially usage of exon 6 in the heart vs. the body.

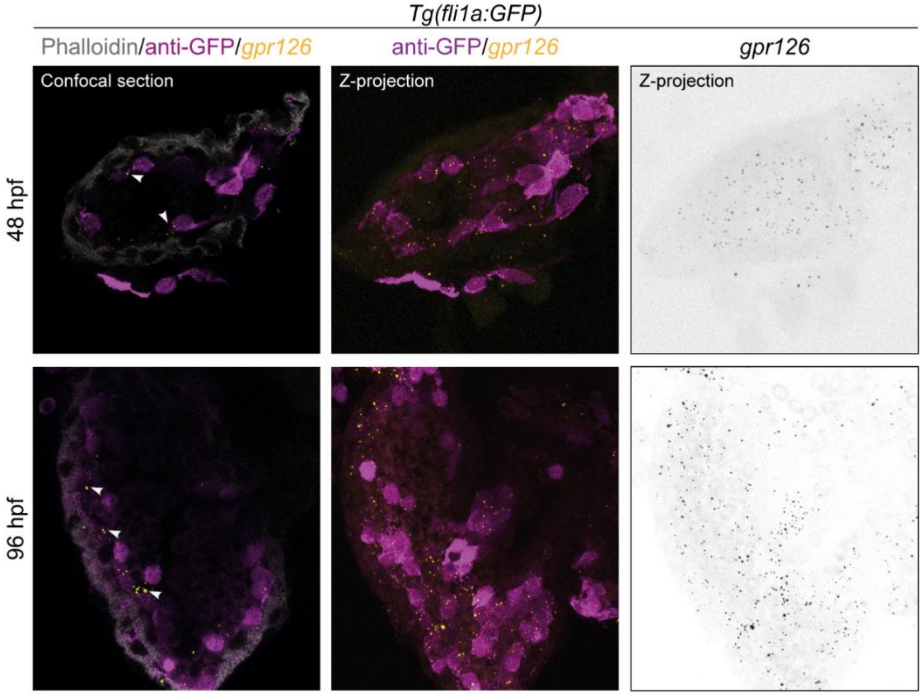

Figure S8. ***gpr126*** **is expressed in the endocardium of zebrafish hearts.**
RNAscope analysis of *gpr126* expression (yellow) in 48 and 96 hours post fertilization (hpf) *Tg(fli1a:GFP)* embryos combined with whole mount phalloidin (myocardium: grey) and anti-GFP (endocardium: magenta) immunostaining. The black dotty signal in the black and white Z-projection image represents *gpr126* expression. Arrows: *gpr126* expression. *n* = 4 biological replicates.

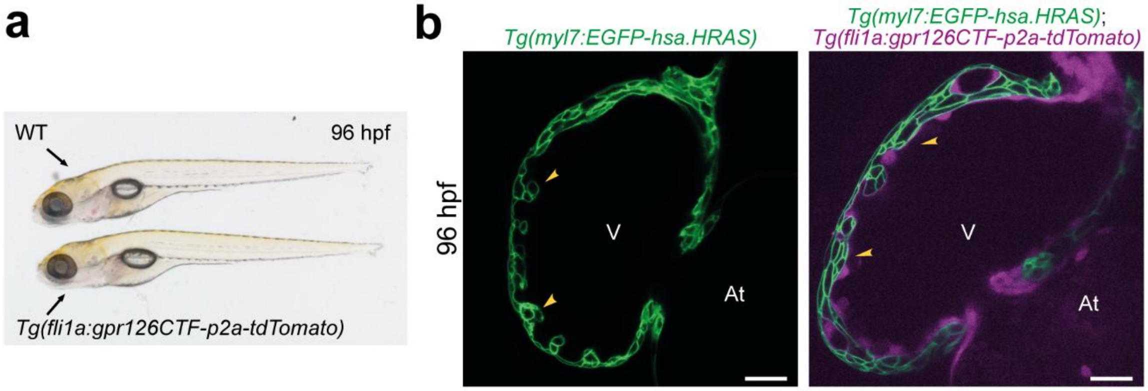

Figure S9. Endocardial CTF overexpression leads to no obvious heart defects.
(a) Representative gross morphology of 96 hours post fertilization (hpf) WT and *Tg(fli1a:gpr126CTF-p2a-tdTomato)* larvae. *n* = 3 independent experiments.
(b) Confocal images (mid-sagittal sections) of *Tg(my7:EGFP-hsa.HRAS)* and *Tg(my7:EGFP-hsa.HRAS)*; *Tg(fli1a:gpr126CTF-p2a-tdTomato)* at 96 hpf. (*n* = 10 and *n* =12, respectively). Arrowheads: trabeculae; V: ventricle; At: atrium; Scale bars: 20 µm.

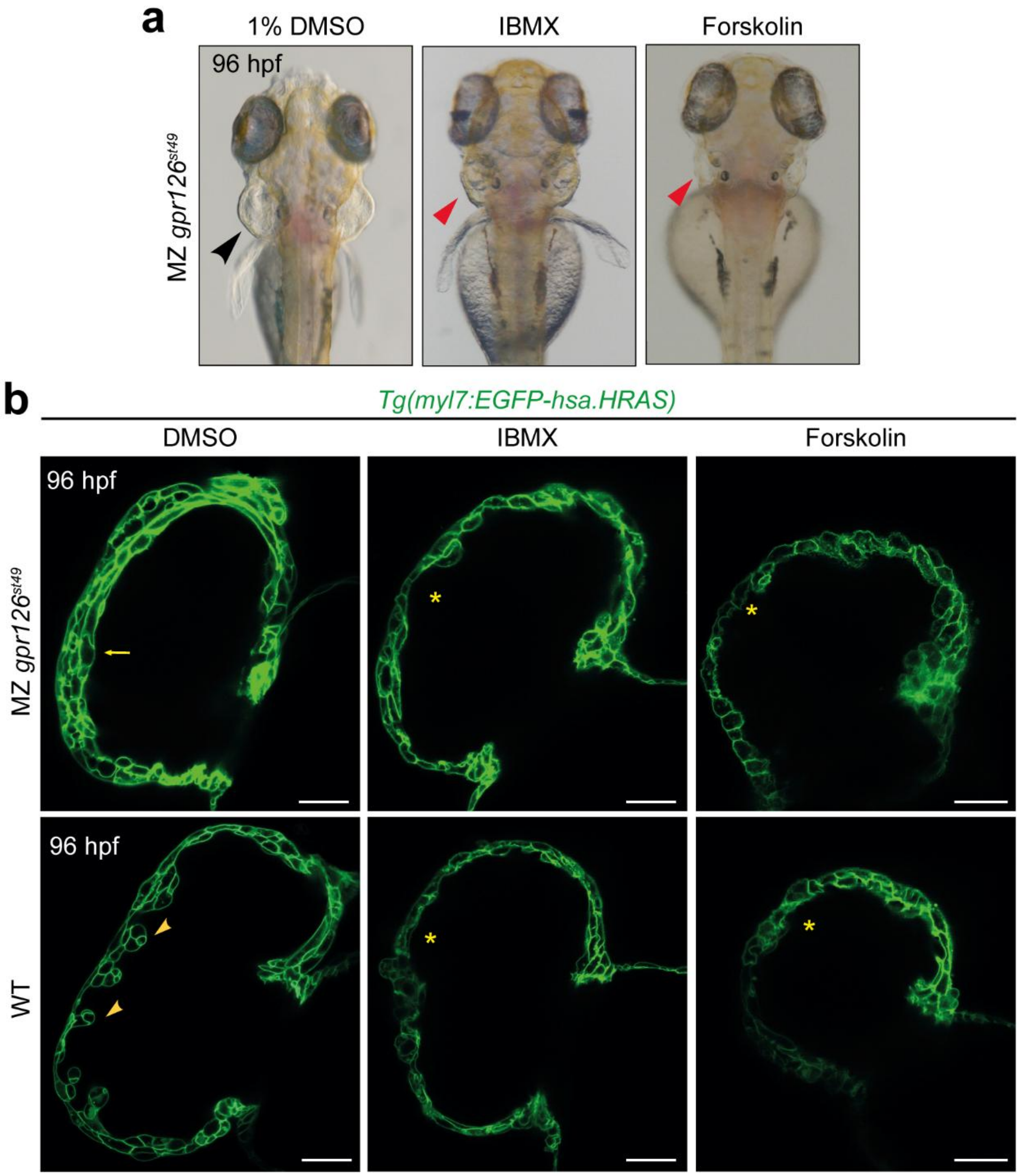

Figure S10. Forskolin and IBMX treatment rescues multilayering in MZ *gpr126^{st49}* hearts but impairs trabeculation in WT hearts.

(a) Dorsal views of 120 hours post fertilization (hpf) maternal zygotic (MZ) *gpr126^{st49}* larvae treated with control, IBMX and forskolin. Black arrowhead: puffy ear; red arrowhead: rescued ear phenotype.

(b) Confocal images (mid-sagittal sections) of 96 hpf *Tg(my7:EGFP-hsa.HRAS)*; WT and MZ *gpr126^{st49}* hearts treated with 1% DMSO or IBMX or forskolin. *n* = 10/treatment in 3 independent experiments. Arrows: multilayered ventricular wall; arrowheads: trabeculae; asterisks: absence of trabeculae.

Scale bars: 20 µm.

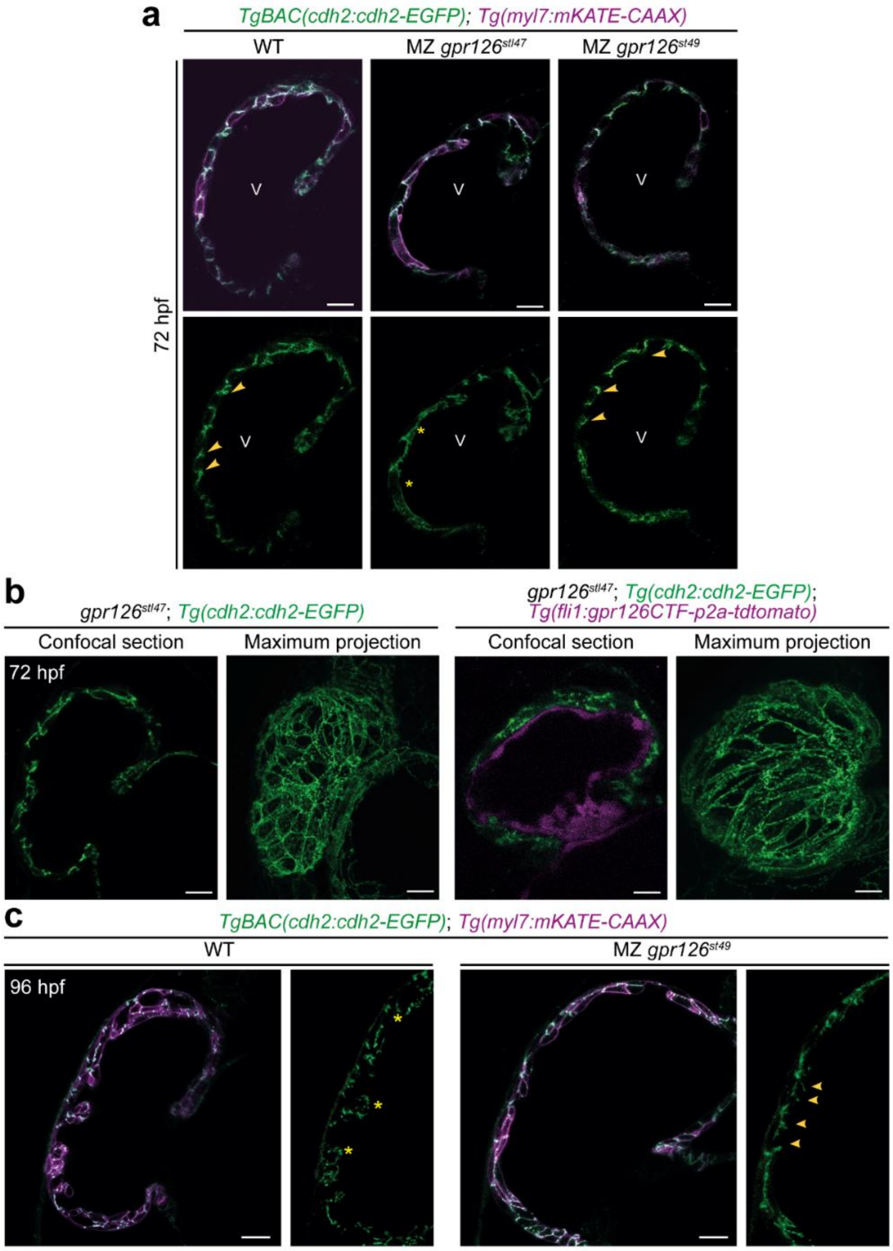

Figure S11. CTF overexpression in MZ *gpr126^{stl47}* mutants does not rescue compact wall integrity defects.

(a) Representative confocal images (mid-sagittal sections) of *TgBAC(cdh2:cdh2-EGFP)*; *Tg(myl7:mKATE-CAAX);*WT, maternal zygotic (MZ) *gpr126^{stl47}*, and MZ *gpr126^{st49}* hearts at 72 hours post fertilization (hpf). Arrowheads: lateral N-cadherin localization. Asterisks: N-cadherin mislocalization. V: ventricle. Scale bars: 20 µm.

(b) Confocal images and maximum projection of 72 hpf *gpr126^{stl47}; Tg(Cdh2:Cdh2-EGFP)* and *gpr126^{stl47};Tg(Cdh2:Cdh2-EGFP)*; *Tg(fli1:gpr126CTF-p2a-tdTomato)* hearts (*n* = 6 and *n* = 8, respectively). Scale bars: 20 µm.

(c) Representative confocal images (mid-sagittal sections) of 96 hpf *TgBAC(cdh2:cdh2-EGFP);(myl7:mKATE-CAAX);*WT and MZ *gpr126^{st49}* hearts. Asterisks: punctate distribution of N-cadherin around cardiomyocytes. Arrowheads: lateral N-cadherin distribution. V: ventricle, At: atrium. Scale bars: 20 µm.

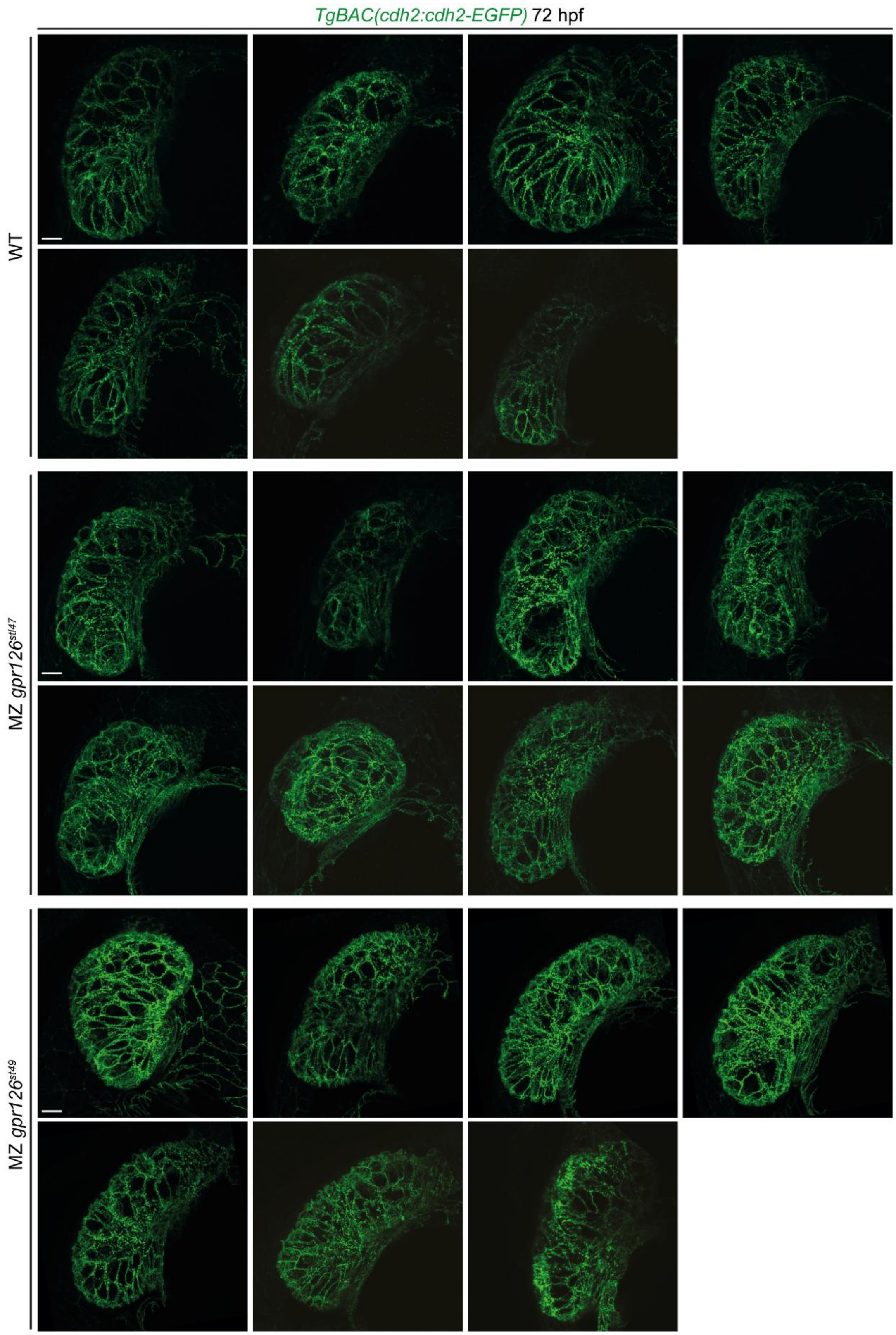

Figure S12. MZ *gpr126^{stl47}* mutants have disorganized adherens junctions.
Maximum intensity projections of *TgBAC(cdh2:cdh2-GFP)*; WT, maternal zygotic (MZ) *gpr126^{stl47}*, and MZ *gpr126^{st49}* hearts at 72 hours post fertilization (hpf).

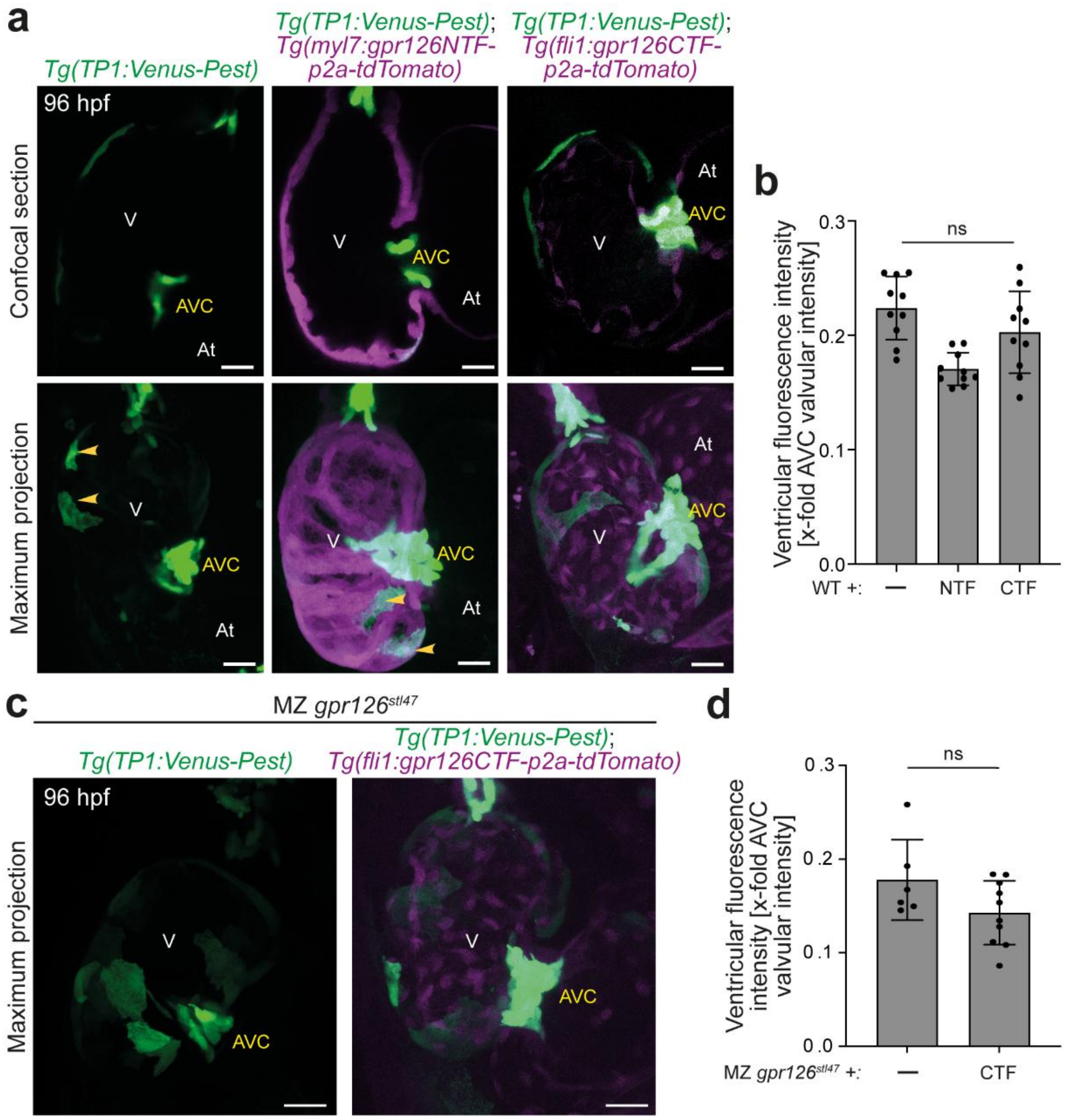

Figure S13. NTF and CTF overexpression in WT embryos and CTF overexpression in MZ *gpr126*^{stl47} mutants does not lead to change in myocardial Notch activity.

(a) Confocal section and maximum projections of representative *Tg(TP1:Venus-PEST)*, *Tg(TP1:Venus-PEST)*; *Tg(myl7:gpr126NTF-p2a-tdTomato)* and *Tg(TP1:Venus-PEST); Tg(fli1:gpr126CTF-p2a-tdTomato)* hearts at 96 hours post fertilization (hpf). Yellow arrows: Notch activity. AVC: atrioventricular canal. V: ventricle. At: atrium. Scale bars: 20 µm.

(b) Quantification of a. n = 10. Each point represents a heart. WT vs. WT + NTF: p = 0.4671. WT vs. WT + CTF: p = 0.7693 (one way ANOVA with Dunnett's multiple comparisons test). Data are mean ± S.D. ns: not statistically significant.

(c) Maximum intensity projections of representative maternal zygotic (MZ) *gpr126*^{stl47}; *Tg(TP1:Venus-PEST),* and MZ *gpr126*^{stl47}*;Tg(TP1:Venus-PEST)*; *Tg(fli1:gpr126CTF-p2a-tdTomato)* hearts (n = 6 and n = 10, respectively) at 96 hpf. Scale bars: 20 µm.

(d) Quantification of c. Each point represents a heart. p = 0.0918 (two-tailed unpaired Student's t-test). Data are mean ± S.D. ns: not statistically significant.

Table S1: Exon counting bin (B) defined by DEXSeq for the gene *adgrg6*

<table>
<tr><th rowspan="2">Exon Counting Bin</th><th rowspan="2">Start</th><th rowspan="2">End</th><th rowspan="2">Length</th><th colspan="4">Exon Number</th></tr>
<tr><th>adgrg6-204 (canonical isoform)</th><th>adgrg6-201</th><th>adgrg6-202</th><th>adgrg6-203</th></tr>
<tr><td>B001</td><td>37383034</td><td>37383287</td><td>254</td><td>1</td><td>--</td><td>--</td><td>1</td></tr>
<tr><td>B002</td><td>37393134</td><td>37393135</td><td>2</td><td>--</td><td rowspan="2">1</td><td>--</td><td>--</td></tr>
<tr><td>B003</td><td>37393136</td><td>37393224</td><td>89</td><td>2</td><td>--</td><td>2</td></tr>
<tr><td>B004</td><td>37408147</td><td>37408500</td><td>354</td><td>3</td><td>2</td><td>--</td><td>--</td></tr>
<tr><td>B005</td><td>37413795</td><td>37413967</td><td>173</td><td rowspan="2">4</td><td rowspan="2">3</td><td>--</td><td>3</td></tr>
<tr><td>B006</td><td>37413968</td><td>37414415</td><td>448</td><td>--</td><td>--</td></tr>
<tr><td>B007</td><td>37423014</td><td>37423076</td><td>63</td><td>5</td><td>4</td><td>--</td><td>--</td></tr>
<tr><td>B008</td><td>37426356</td><td>37426424</td><td>69</td><td>6</td><td>5</td><td>--</td><td>--</td></tr>
<tr><td>B009</td><td>37436945</td><td>37437027</td><td>83</td><td>7</td><td>6</td><td>--</td><td>--</td></tr>
<tr><td>B010</td><td>37437172</td><td>37437224</td><td>53</td><td>8</td><td>7</td><td>--</td><td>--</td></tr>
<tr><td>B011</td><td>37437308</td><td>37437349</td><td>42</td><td>9</td><td>8</td><td>--</td><td>--</td></tr>
<tr><td>B012</td><td>37437775</td><td>37437914</td><td>140</td><td>10</td><td>9</td><td>--</td><td>--</td></tr>
<tr><td>B013</td><td>37439414</td><td>37439528</td><td>115</td><td>11</td><td>10</td><td>--</td><td>--</td></tr>
<tr><td>B014</td><td>37439669</td><td>37439757</td><td>89</td><td>12</td><td>11</td><td>--</td><td>--</td></tr>
<tr><td>B015</td><td>37439836</td><td>37440037</td><td>202</td><td>13</td><td>12</td><td>--</td><td>--</td></tr>
<tr><td>B016</td><td>37442366</td><td>37442531</td><td>166</td><td>14</td><td>13</td><td>--</td><td>--</td></tr>
<tr><td>B017</td><td>37443598</td><td>37443738</td><td>141</td><td>15</td><td>14</td><td>--</td><td>--</td></tr>
<tr><td>B018</td><td>37447817</td><td>37447936</td><td>120</td><td>16</td><td>15</td><td>--</td><td>--</td></tr>
<tr><td>B019</td><td>37448605</td><td>37448650</td><td>46</td><td>17</td><td>16</td><td>--</td><td>--</td></tr>
<tr><td>B020</td><td>37448753</td><td>37448859</td><td>107</td><td>18</td><td>17</td><td>--</td><td>--</td></tr>
<tr><td>B021</td><td>37451748</td><td>37451875</td><td>128</td><td>19</td><td>18</td><td>--</td><td>--</td></tr>
<tr><td>B022</td><td>37454428</td><td>37454696</td><td>269</td><td>20</td><td>19</td><td>--</td><td>--</td></tr>
<tr><td>B023</td><td>37454816</td><td>37454918</td><td>103</td><td>21</td><td>20</td><td>--</td><td>--</td></tr>
<tr><td>B024</td><td>37457407</td><td>37457641</td><td>235</td><td rowspan="2">22</td><td rowspan="2">21</td><td>--</td><td>--</td></tr>
<tr><td>B025</td><td>37457642</td><td>37457690</td><td>49</td><td>1</td><td>--</td></tr>
<tr><td>B026</td><td>37459110</td><td>37459211</td><td>102</td><td>23</td><td>22</td><td>2</td><td>--</td></tr>
<tr><td>B027</td><td>37460218</td><td>37460370</td><td>153</td><td>24</td><td>23</td><td>3</td><td>--</td></tr>
<tr><td>B028</td><td>37463293</td><td>37463309</td><td>17</td><td>--</td><td>24</td><td rowspan="2">4</td><td>--</td></tr>
<tr><td>B029</td><td>37463310</td><td>37463331</td><td>22</td><td>--</td><td>--</td><td>--</td></tr>
<tr><td>B030</td><td>37469188</td><td>37469640</td><td>453</td><td>25</td><td>--</td><td>5</td><td>--</td></tr>
</table>

For each exon counting bin (B) defined by DEXSeq for the gene *adgrg6* the table reports: its genomic starting and ending position, its length, and, for all transcript isoforms (adgrg6-204, adgrg6-201, adgrg6-202, and adgrg6-203), the number of the exon to which the exon counting bin belongs.

Table S2: Utilized primers

Primer	Forward primer (5'-3')	Reverse Primer (5'-3')
gpr126 qPCR	TGTCGTTGACTGGGATCATT	CGGTTCCTGGTGAAAGAGTT
myl7 qPCR	GGCTCTTCCAATGTCTTCTCC	GGACTCCAGCTCTTCATCAC
kdrl qPCR	TCTTCACTCTTCACGTGCTTTTTAG	GAAGGTGTGTATCTCCATCAGGAA
rpl13a qPCR	TCTGGAGGACTGTAAGAGGTATGC	AGACGCACAATCTTGAGAGCAG
stl47 genotyping	GTCTTTGTCTCTGTCGATGC	GCTTGTAACTGATATGGAAGCC
st49 genotyping	CTCAACCTAAAACACAAGTGGG	GTGCAATACTGCATTTAACAC
stl47 site directed mutagenesis	TCCTGCAAAGTCCATACAGGCCCC GGCTGGC	CCTGTATGGACTTTGCAGGACTGG CTGGGTGG
st49 site directed mutagenesis	CATTTGGAGTAACAGAAAGATCCG AATC	TCTTTCTGTTACTCCAAATGTGCAA TCTC
plenti amplification	GTACAAGTGAGATATCCAGCACAG TGGC	ACGAAATCATTCTGACGGTTCACTA AAC
Gpr126 amplification	AACCGTCAGAATGATTTCGTTCAT CAGTG	GCTGGATATCTCACTTGTACAGCTC ATC
B004F-B007R	TCCACTGGAAATGTGATGGA	CCAAGTCCAGAAGCACAACC
B007F-B011R	GCAGGTTGTGCTTCTGGACT	CGTGTGGTTGTGATCCTTGA
