# Supplementary material for: Adgrg6/Gpr126 is required for compact wall integrity and establishing trabecular identity during cardiac trabeculation
Source: Nat Commun. 2026 Feb 7;17:1484. doi: 10.1038/s41467-026-69292-5 (PMC12886893; doi:10.1038/s41467-026-69292-5)
Supplement: Supplementary file 3 — Reporting Summary [file 41467_2026_69292_MOESM3_ESM.pdf]

nature portfolio

Corresponding author(s): Felix Engel

Last updated by author(s): Dec 27, 2025

Reporting Summary

Nature Portfolio wishes to improve the reproducibility of the work that we publish. This form provides structure for consistency and transparency in reporting. For further information on Nature Portfolio policies, see our Editorial Policies and the Editorial Policy Checklist.

Statistics

For all statistical analyses, confirm that the following items are present in the figure legend, table legend, main text, or Methods section.

n/a	Confirmed	
☐	☒	The exact sample size (n) for each experimental group/condition, given as a discrete number and unit of measurement
☐	☒	A statement on whether measurements were taken from distinct samples or whether the same sample was measured repeatedly
☐	☒	The statistical test(s) used AND whether they are one- or two-sided<br>*Only common tests should be described solely by name; describe more complex techniques in the Methods section.*
☒	☐	A description of all covariates tested
☐	☒	A description of any assumptions or corrections, such as tests of normality and adjustment for multiple comparisons
☐	☒	A full description of the statistical parameters including central tendency (e.g. means) or other basic estimates (e.g. regression coefficient) AND variation (e.g. standard deviation) or associated estimates of uncertainty (e.g. confidence intervals)
☐	☒	For null hypothesis testing, the test statistic (e.g. F, t, r) with confidence intervals, effect sizes, degrees of freedom and P value noted<br>*Give P values as exact values whenever suitable.*
☒	☐	For Bayesian analysis, information on the choice of priors and Markov chain Monte Carlo settings
☒	☐	For hierarchical and complex designs, identification of the appropriate level for tests and full reporting of outcomes
☒	☐	Estimates of effect sizes (e.g. Cohen's d, Pearson's r), indicating how they were calculated

Our web collection on statistics for biologists contains articles on many of the points above.

Software and code

Policy information about availability of computer code

Data collection	Immunostaining was detected with Carl Zeiss 800 confocal microscope with ZEN blue software. Bright field images of zebrafish larvae and in-situ hybridization images were obtained by Carl Zeiss AG Axiozoom V16 stereoscope with ZEN blue software.<br>qPCR was performed with CFX Connect™ Real-Time PCR (Bio-Rad).<br>Western blot signal was detected with iBright 1500 (Thermo Fischer Scientific).<br>Serial Cloner(2.6.1) and NEBuilder were used for in-silico design for assembly cloning.
Data analysis	ImageJ (National Institutes of Health)/Fiji were used for image data analysis. 3D surface rendering was performed using IMARIS 9.0 x64 (Bitplane). GraphPad Prism (version 8.3.0 and 10.2.0) was used for statistical analysis.

For manuscripts utilizing custom algorithms or software that are central to the research but not yet described in published literature, software must be made available to editors and reviewers. We strongly encourage code deposition in a community repository (e.g. GitHub). See the Nature Portfolio guidelines for submitting code & software for further information.

Data

Policy information about availability of data

All manuscripts must include a data availability statement. This statement should provide the following information, where applicable:

- Accession codes, unique identifiers, or web links for publicly available datasets
- A description of any restrictions on data availability
- For clinical datasets or third party data, please ensure that the statement adheres to our policy

The sequencing data generated in this study have been deposited in the European Nucleotide Archive (ENA) at EMBL-EBI under accession number PRJEB98567 (https://www.ebi.ac.uk/ena/browser/view/PRJEB98567) and PRJEB98842 (https://www.ebi.ac.uk/ena/browser/view/PRJEB98842)". Source data are provided as Supporting Information.

Research involving human participants, their data, or biological material

Policy information about studies with human participants or human data. See also policy information about sex, gender (identity/presentation), and sexual orientation and race, ethnicity and racism.

Reporting on sex and gender	*Use the terms sex (biological attribute) and gender (shaped by social and cultural circumstances) carefully in order to avoid confusing both terms. Indicate if findings apply to only one sex or gender; describe whether sex and gender were considered in study design; whether sex and/or gender was determined based on self-reporting or assigned and methods used.*<br>*Provide in the source data disaggregated sex and gender data, where this information has been collected, and if consent has been obtained for sharing of individual-level data; provide overall numbers in this Reporting Summary. Please state if this information has not been collected.*<br>*Report sex- and gender-based analyses where performed, justify reasons for lack of sex- and gender-based analysis.*
Reporting on race, ethnicity, or other socially relevant groupings	*Please specify the socially constructed or socially relevant categorization variable(s) used in your manuscript and explain why they were used. Please note that such variables should not be used as proxies for other socially constructed/relevant variables (for example, race or ethnicity should not be used as a proxy for socioeconomic status).*<br>*Provide clear definitions of the relevant terms used, how they were provided (by the participants/respondents, the researchers, or third parties), and the method(s) used to classify people into the different categories (e.g. self-report, census or administrative data, social media data, etc.)*<br>*Please provide details about how you controlled for confounding variables in your analyses.*
Population characteristics	*Describe the covariate-relevant population characteristics of the human research participants (e.g. age, genotypic information, past and current diagnosis and treatment categories). If you filled out the behavioural & social sciences study design questions and have nothing to add here, write "See above."*
Recruitment	*Describe how participants were recruited. Outline any potential self-selection bias or other biases that may be present and how these are likely to impact results.*
Ethics oversight	*Identify the organization(s) that approved the study protocol.*

Note that full information on the approval of the study protocol must also be provided in the manuscript.

Field-specific reporting

Please select the one below that is the best fit for your research. If you are not sure, read the appropriate sections before making your selection.

☒ Life sciences ☐ Behavioural & social sciences ☐ Ecological, evolutionary & environmental sciences

For a reference copy of the document with all sections, see nature.com/documents/nr-reporting-summary-flat.pdf

Life sciences study design

All studies must disclose on these points even when the disclosure is negative.

Sample size	No sample size calculation was performed. Sample size was chosen based on previous similar studies and our experience.
Data exclusions	No data were excluded from the study.
Replication	All live imaging, immunostainings, rescue experiments and drug treatments were performed in at least 3 independent biological replicates. All attempts at replication were successful. RT-qPCR analysis from sorted cardiomyocytes and endocardial cells (for gpr126 expression analysis) was performed in 2 independent biological replicates.
Randomization	Sample allocation was random. For zebrafish experiments, a subset of embryos per clutch was randomly chosen for each experiment. For all phenotypic characterizations, larvae were randomly chosen among each genotype population.
Blinding	There was no systematic blinding set up (mutants can be identified by puffy ears). However, several researchers from different laboratories performed independently different experiments of the study whereby the results verified the data of each other.

Reporting for specific materials, systems and methods

We require information from authors about some types of materials, experimental systems and methods used in many studies. Here, indicate whether each material, system or method listed is relevant to your study. If you are not sure if a list item applies to your research, read the appropriate section before selecting a response.

Materials & experimental systems

n/a	Involved in the study
☐	☒ Antibodies
☐	☒ Eukaryotic cell lines
☒	☐ Palaeontology and archaeology
☐	☒ Animals and other organisms
☒	☐ Clinical data
☒	☐ Dual use research of concern
☒	☐ Plants

Methods

n/a	Involved in the study
☒	☐ ChIP-seq
☐	☒ Flow cytometry
☒	☐ MRI-based neuroimaging

Antibodies

Antibodies used	Antibodies for immunostaining and Western blot:<br>Primary antibodies:<br>Mouse anti-GFP (Abcam, ab38689) (1:500)<br>Mouse anti-FLAG M2(Sigma Aldrich, F3165) (1:500)<br>Rabbit anti-HA (Abcam, ab9110) (1:500)<br><br>Secondary antibodies:<br>donkey anti-mouse/rabbit conjugated to Alexa Fluor -488, -594, and -647 (Life Technologies) (1:500)<br>HRP-conjugated secondary antibodies: Donkey anti-rabbit (GE Healthcare, NA934), Sheep anti-mouse (GE Healthcare, NA931) (1:10,000)<br><br>Antibodies for in-situ hybridization:<br>Anti-digoxigenin-AP (alkaline phosphatase) antibody (Fab fragments, Roche #11093274910)
Validation	All primary and secondary antibodies used in the study were commercial and used according to the profile of manufactures. All other details are provided in the methods section.

Eukaryotic cell lines

Policy information about cell lines and Sex and Gender in Research

Cell line source(s)	ARPE-19 (CRL-2302) cells and HEK293T (CRL-2316) cells were obtained from ATCC.
Authentication	None of the cell lines were authenticated.
Mycoplasma contamination	All used cell lines are tested negative for mycoplasma.
Commonly misidentified lines<br>(See ICLAC register)	None

Animals and other research organisms

Policy information about studies involving animals; ARRIVE guidelines recommended for reporting animal research, and Sex and Gender in Research

Laboratory animals	Danio rerio (Zebrafish) of both sexes were used in this study.<br>Adult zebrafish were used for mating and experimental zebrafish larvae were analyzed till 5 days post fertilization (dpf).<br>The following strains were used in this study: Tg(myl7:EGFP-hsa.HRAS)s88371, Tg(-0.2myl7:EGFP-podocalyxin)bns1041, Tg (myl7:mKate-CAAX)sd11, TgBAC(cdh2:cdh2-eGFP,crybb1:ECFP)zf517, Tg(EPV.Tp1-Mmu.Hbb:Venus-Mmu.Odc1)s940, , gpr126stl47, gpr126st49, Tg(krt4:GFP)sqet33, Tg(fli1:gpr126-p2a-tdTomato), Tg(myl7:gpr126NTF-p2a-tdTomato), and Tg(fli1:tdTomato-p2a-gpr126CTF).
Wild animals	No wild animals were used in this study.
Reporting on sex	Sex was not a part of the study design. The generation of maternal zygotic progeny is described in the methods section.

Field-collected samples	The study did not involve samples collected from the field.
Ethics oversight	All procedures performed on animals conform to the guidelines from Directive 2010/63/EU of the European parliament on the protection of animals used for scientific purposes and were approved by the Animal protection committee (Tierschutzkommision) of the REGIERUNG VON UNTERFRANKEN (local government) under the protocols AZ. 55.2.2-2532.2-1145-13 and AZ.I/39-1/FN003.

Note that full information on the approval of the study protocol must also be provided in the manuscript.

Plants

Seed stocks	*Report on the source of all seed stocks or other plant material used. If applicable, state the seed stock centre and catalogue number. If plant specimens were collected from the field, describe the collection location, date and sampling procedures.*
Novel plant genotypes	*Describe the methods by which all novel plant genotypes were produced. This includes those generated by transgenic approaches, gene editing, chemical/radiation-based mutagenesis and hybridization. For transgenic lines, describe the transformation method, the number of independent lines analyzed and the generation upon which experiments were performed. For gene-edited lines, describe the editor used, the endogenous sequence targeted for editing, the targeting guide RNA sequence (if applicable) and how the editor was applied.*
Authentication	*Describe any authentication procedures for each seed stock used or novel genotype generated. Describe any experiments used to assess the effect of a mutation and, where applicable, how potential secondary effects (e.g. second site T-DNA insertions, mosiacism, off-target gene editing) were examined.*

Flow Cytometry

Plots

Confirm that:

☐ The axis labels state the marker and fluorochrome used (e.g. CD4-FITC).

☐ The axis scales are clearly visible. Include numbers along axes only for bottom left plot of group (a 'group' is an analysis of identical markers).

☐ All plots are contour plots with outliers or pseudocolor plots.

☐ A numerical value for number of cells or percentage (with statistics) is provided.

Methodology

Sample preparation	Larval hearts from Tg(myl7:GFP-CAAX); Tg(kdrl:nls-mCherry)) at 48 and 96 hpf were manually dissected in DMEM+10% FBS, centrifuged for 3 min at 5000 rpm, washed in 1 ml of Hanks' Balanced Salt Solution (HBSS), and dissociated into single cells in 100 µl of Enzyme 1 and 5 µl of Enzyme 2 (Pierce Cardiomyocytes Dissociation Kit, ThermoFisher Scientific) for 30 min at 350 rpm shaking at 30°C. The cells were centrifuged for 5 min at 5000 rpm, and the dissociation media was removed and replaced by DMEM+10% FBS. More than 100 hearts were pooled together for each independent experiment.
Instrument	The cells were sorted using a BD FACSAriaTM III.
Software	The software used for sorting and analysis was BD FACSDiva v8.0.1.
Cell population abundance	*Describe the abundance of the relevant cell populations within post-sort fractions, providing details on the purity of the samples and how it was determined.*
Gating strategy	Cells were obtained from reporter Tg(myl7:EGFP-hsa.HRAS); Tg(kdrl:nls-mCherry) larvae. Genetically fluorescently labeled cells were a clearly distinct population from non-labeled cells, allowing the separation of genetically fluorescently labeled cells from non-labeled cells. RNAscope analysis of gpr126, performed by the Engel laboratory, confirmed the results of the FACS data by the Stainier laboratory, namely the enrichment of gpr126 in the endocardium. Raw .fcs files were removed by the core facility during routine cleanup and were not retained by the lab.

☐ Tick this box to confirm that a figure exemplifying the gating strategy is provided in the Supplementary Information.
